# Performance Comparison Between a Statistical Model, a Deterministic Model, and an Artificial Neural Network Model for Predicting Damage From Pitting Corrosion

**DOI:** 10.6028/jres.099.047

**Published:** 1994

**Authors:** M. Urquidi-Macdonald, D. D. Macdonald

**Affiliations:** Pennsylvania State University, University Park, PA 16802

**Keywords:** artificial neural networks, deterministic, mathematical modeling, pitting corrosion, statistics

## Abstract

Various attempts have been made to develop models for predicting the development of damage in metals and alloys due to pitting corrosion. These models may be divided into two classes: the empirical approach which employs extreme value statistics, and the deterministic approach based on perceived mechanisms for nucleation and growth of damage. More recently, Artificial Neural Networks (ANNs), a nondeterministic type of model, has been developed to describe the progression of damage due to pitting corrosion. We compare the three approaches above-statistical, deterministic, and neural networks. Our goal is to illustrate the advantages and disadvantages of each approach, in order that the most reliable methods may be employed in future algorithms for predicting pitting damage functions for engineering structures. To illustrale the difficulty that we face in predicting cumulative pitting damage, we selected a set of data that was collected in the laboratory. We compare and contrast the three approaches by reference to this data set.

## 1. Introduction

On the basis of laboratory studies [1], and through the analysis of field data collected over the past decade by Battelle Columbus Laboratory [2], several factors have been identified as contributing to the development of pitting damage in gas fired heat exchangers in domestic and industrial service:

- The type of alloy used for fabricating the heat exchanger
- Chloride concentration in the flue gas condensate
- Temperature
- Exposure time
- Ambient versus indoor air
- pH
- Electrochemical potential

Unfortunately, few of these factors are simply related to the damage functions or to one another. Accordingly, it is seldom possible to establish a simple empirical equation for predicting pitting damage as a function of these variables. The case cited above is not atypical, and it illustrates the difficulties faced by those who seek to develop predictive models for assessing corrosion damage. Indeed, the data base established by Battelle is probably one of the best that currently exists for the development of pitting damage in an industrial system. A full interpretation of the Battelle data in terms of statistical, deterministic, and artificial neural network models is published elsewhere [3].

In the present paper, we use a more restricted database to illustrate how various classes of models arc used to analyze the damage caused by pitting corrosion. These models include a statistical approach based on the Weibull distribution function, a deterministic model based on a physicochemical mechanism, and an Artificial Neural Network (ANN) that assumes neither a mathematical model nor a physical model, but which seeks to establish relationships between the dependent and independent variables by examining the patterns contained within the data set.

## 2. Experimental Data

We used laboratory data to illustrate the time and potential dependencies of pitting damage. To do this, we chose a laboratory data set for which the following independent variables were identified: 1) concentration of minor alloy elements in weight percentages, 2) difference in oxidation state between host metal and minor alloy elements, 3) applied potential, and 4) time of observation. Independent variables 1) and 2) are related to the type of alloy; and independent variable 4) together with solution composition (which was maintained constant) is determined by the electrochemical potential. Temperature, solution composition, and pH were maintained constant. The dependent variable was the total number of pits.

We then used this set to illustrate the prediction of cumulative damage for pitting corrosion using three different models: statistical, deterministic, and artificial neural networks. The data were measured by English and Macdonald at SRI International [1].

Several alloys of nickel were fabricated. Each of the alloys tested was arc-melted from powders under an Argon gas blanket in a sealed container. Binary nickel alloys containing Al, Ta, and Mo in nominal concentrations of 0.1%, 0.5%, 1%, 3%, 5%, and 8% by weight were cast as 100 g buttons and were sectioned in an acrylic plastic before polishing. The alloying elements were selected on the basis of their oxidation states relative to nickel (oxidation state = 2). The excess oxidation states range from 1 for Al to 4 for Mo.

The polished specimens were placed in a cell. The electrode potential was swept in the positive direction at 1 mV/s from an initial potential of 0.0 V. This results in a distribution in breakdown potentials. Alternatively, the potential was stepped from 0.0 V to 0.325 V, 0.375 V, 0.4 V, and 0.45 V. This resulted in a distribution in induction (or observation) times for the nucleation of pits.

In both types of experiments the pit nucleation and growth events were photographed at 65 × magnification at regular intervals. The number of pits were counted on the pictures taken at different times and conditions.

The pitting data were measured several times on a similar sample to explore reproducibility. The reproducibility in pure nickel appeared satisfactory (about 10% difference between runs), but the reproducibility from alloy composition to alloy composition was different. Reproducibility was better at high potentials perhaps because the total number of pits developed was higher. Reproducibility appeared to be better at high minor alloy contents and high oxidation states (about 20%), than at low minor alloy contents and low oxidation states (about 50%). Regardless of the poor reproducibility in some of the samples, a general trend was observed: a) The cumulative number of pits diminishes with 1) minor alloy element content, and 2) with increasing difference in oxidation state between the base alloy and the minor alloy element; and b) The cumulative number of pits grows with increasing applied potential and observation time.

Cumulative pitting damage is an irreversible, dynamic, time decay, environmentally related process. The literature is abundant in pitting corrosion data, but there is a lack of good quality data because of the difficulty of measuring pitting corrosion when controlling all the environmental parameters.

All model building is concerned with an attempt to increase our knowledge of complex physical reality. The parameters plus the validity of the model must be determined from the data. The philosophy behind the type of model is different. The information obtained from a purely probabilistic model (statistic and stochastic models) is about finding embodied in the data trends that can be used in future predictions. The information obtained from a deterministic model is about the physical meaning of the phenomena itself. The information obtained with a ANN model is about the dependency and importance of input/output relationships. In any case, the model capabilities need to be tested.

We can start the process of solving our problem by listing facts, listing observations, and listing existing laws relating variables and outputs. Then we have to ask ourselves which will be the best model to describe the problem, and what do we expect from the model. Later we need to identify the model or models to use; specify the constraints, choose the coordinates, and apply the laws dictated by the model. Important questions related to the choice of a correct model are: Is the process static or dynamic? Is the process stationary or not?; Are the available data distributed or not? What do the data mean? What is the data variance? What are the correlations?. In any case, the fitted model you use for analyzing your data is the nearest representation of the true situation you have available.

## 3. Statistical Approach and Resulta

Stochastic processes are dynamic, and good examples are fatigue, wear, and crack or pit growth. There are two main types of stochastic processes: stationary and nonstationary.

It is well known from experimental data that cumulative pitting damage is a nonstationary phenomenon. It is well known that nonstationary models and their estimation are notoriously difficult problems to handle except for special cases. Discrete state continuous Markoff processes are good examples of models that describe nonstationary stochastic phenomena. However, there is no literature on problem solving using nonstationary finite Markoff chains [4]. On the other hand, for the last data set [1] (measured at the laboratory), the cumulative damage versus time was measured, but the pit depth versus number of pits was not. Therefore, it is impossible to derive a dynamic model for pitting damage using that data set. The only option available is to try to fit a static model (i.e., our hypothesis is that the numbers of pits versus pit depth does not change with time). We choose a 2 parameter Weibull distribution; for which we assume that the independent parameters are potential, and the oxidation state and concentration of the minor alloying elements. The dependent variables are the cumulative number of pits and the induction time. The Weibull distribution function is

$$F\left(x\right)=(1-\exp(-\;{\left(x/\beta \right)}^{\alpha }))$$

where *α* and *β* are fitting parameters and *x* is the dependent variable of interest.

We normalized the data set to 80% of its maximum value, allowing 20% of the pits to nucleate if the time would have been extended to infinity. For each potential, oxidation state, and percentile of minor alloy element, we performed a nonlinear fit to estimate the Weibull fitting parameters.

The choice of a Weibull distribution is arbinary, we chose a Weibull distribution instead of some other probabilistic distribution because of the flexibility that this distribution offers in filling different shapes obtained when plotting cumulative damage versus dependent variables.

**Fig. 1:**
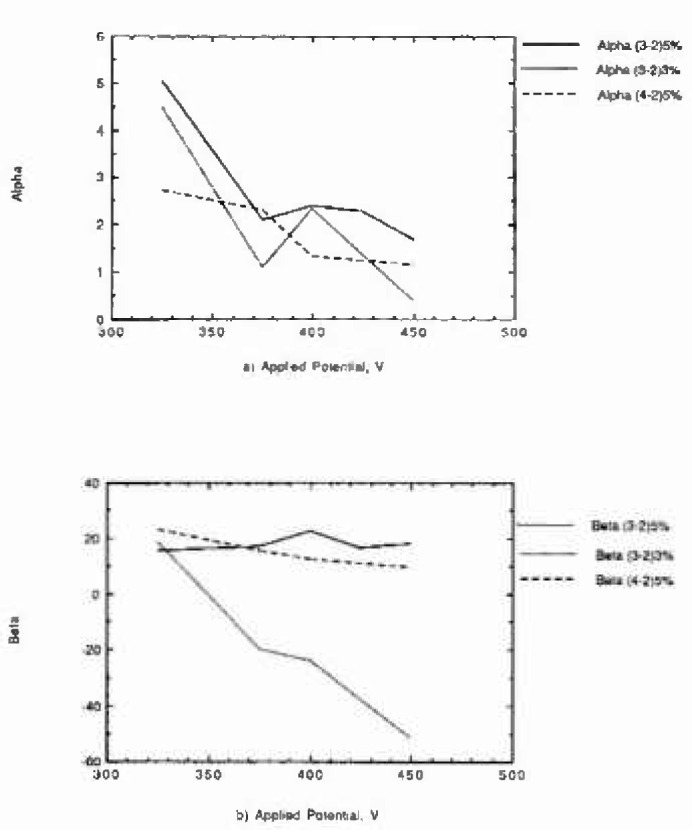
(a) Alpha parameter of the Weibull distribution versus applied potential; at several percentiles of minor alloying elements, and oxidation stales. (b) Beta parameter of the Weibull distribution versus applied potentini; at several percentiles of minor alloying elements, and oxidation states.

The nonlinear fits were acceptable (sum of square errors between fit and data <20% for *α* or *β*). We used those data sets for which smooth changes of *α* and *β* were calculated as a function of potential. Than reduced the data base to about 50% of the total available (the total data base had 1400 data lines containing number of pits at different observation times, applied potential, oxidation states, and percemik of minor elements). We plotted the *α* and *β* values as a function of potential. Figures 1a and 1b show the results. The beta parameter of the Weibull distribution appears to not change with applied potential at high concentration of minor alloy elements (5%), but it changes drastically with applied potential at low concentrations of minor alloy element (3%, 1%). We then fit polynomials describing *α* and *β* as functions of applied potential, oxidation state, and percentile of minor alloy elements.

**Fig. 2:**
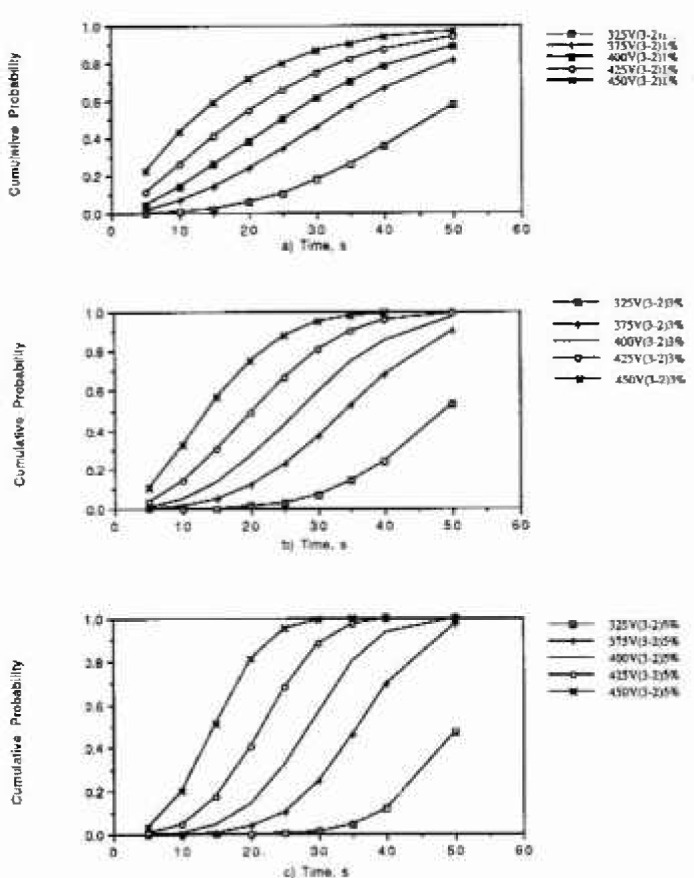
Predicted cumulative probability, obtained using the statistical model, versus time of observation, at several applied potentials, (a) Oxidation state 3-2; and 1% of minor alloying clement segregated in the film, (b) Oxidation state 3-2; and 3% of minor alloying element segregated in the film, (c) Oxidation state 3-2; and 5% of minor alloying clement segregated in the film.

The Weibull distribution with *α* and *β* as parameters was used to generate the cumulative damage function Figures 2a, b, and c show the predictions obtained with thus statistical model. When we compared the. predictions obtained with this model and the measured data, we observed that both trends are similar, However, it would be very risky to use the model to make predictions for other oxidations states, percentile of minor alloys elements, or applied potentials outside the range for which the Weibull-parameters were calculated.

It is well known that the Weibull distribution is a sufficiently flexible function that practically any set of data can be filled by it. However, the problem we faced is that we do not know *a priori* the correct relationships between *α* and *β* and the independent variables.

Predictions with the same model for oxidation stales greater than 3-2 gave cumulative probability of zero at any time and are not shown. The designation “3-2” referes to the oxidation state of the alloying element (Al = 3) and the host metal (Ni = 2).

## 4. Deterministic Model

A completely successful model must account for all of the phenomenological correlations that exist between pitting susceptibility and pit velocity, and various environmental and electrochemical factors, such as temperature, pH, [Cl], potential, time, and alloy composition. The Point Defect Model (PDM) [5, 6] accounts for the effects of electrochemical potential, alloy composition, and environmental conditions on the nucleation of pits.

The determmistic model is based on the PDM and the Solute Vacancy Interaction Model (SVIM) [7–10]. The PDM proposes that passivity breakdown occurs because of an enhanced flux of cation vacancies front the film/solution to the metal/film interface. If the excess of vacancies arriving at the interface between the metal and the film can not be absorbed into the metal or be annihilated by some appropriate mechanism at high enough rate, they accumulate to form a vacancy condensate at the metal film/interface, which then grows to a critical size. The PDM is used to calculate the breakdown potential and induction time. The effect of the minor alloying elements in the oxide film on the breakdown parameters is modeled using the SVIM. The SVIM is based on the hypothesis that highly oxidized solutes in the passive film electrostatically complex with the mobile cation vacancies.

The PDM and SVIM results in distributed values of the breakdown potential and induction time, and complexing between the immobile alloying element in the film (the “solute”) and the mobile vacancies diminishes the flux of vacancies across the film. This leads to an increase in the breakdown potential and the induction time for film breakdown. The higher the net oxidation state (minor alloy element oxidation-host ion oxidation) and/or the higher the percentile of minor alloying elements in the film, the greater the effect on reducing the flux of vacancies and hence in increasing the pitting potential and the induction time. Once the pits nucleate, they grow at different rates. To calculate the pit growth rate we used 1) a simplistic steady state model suggested by Alkire [11]; and 2) a nonstationary model developed by us [12–13]. The stationary model is expected to be adequate for only short times.

The overall model (combination of the PDM, SVIM, and pit growth) requires the defining of a number of parameters, as shown in Table 1.

Figure 3a shows the cumulative probability of the number of pits (normalized to 1) as a function of pit depth and observational time of 50 s, for an applied voltage of 0.325 V and for several concentrations of the minor alloying element with oxidation state of 3-2 (example aluminum in nickel). Figures 3b and 3c show similar plots for oxidation states of 4-2 (e.g., titanium-nickel) and 6-2 (e.g., molybdenum-nickel), respectively, for the same conditions. It is interesting to note the great effect of minor alloying elements with high oxidation states. The model predicts that the cumulative probability of the number of pits at all pit depths is higher at lower minor alloying element oxidation state and at lower concentration of the minor alloying element. Because the model does not assume a total number of breakdown sites only a normalized probability is obtained.

**Fig. 4:**
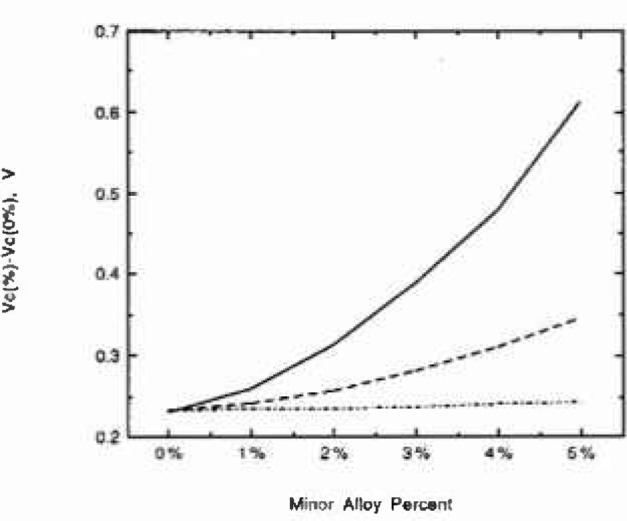
Difference between calculated breakdown potential of nickel containing 0%−5% of minor alloying elements with oxidation states of (____3-2: Ni-AI), (---4-2: Ti-Ni), and (-.-.-.6-2: Mo-Ni) and calculated breakdown potential of pure nickel (containing 0% of minor alloying elements).

Figure 4 shows the beneficial effect of adding minor alloying elements with high oxidation states. The breakdown potential is shifted in the positive direction, indicating that higher potentials are necessary to achieve the same damage.

## 5. Artificial Neural Network Model and Results

Probably the most efficient method, when data are available, of establishing relationships between inputs and results is to use artificial intelligence techniques. Accordingly, we describe here an Artificial Neural Network (ANN) for predicting pitting damage functions for condensing heat exchangers. When the net is trained with reliable data and knowledge, we are able to accurately predict damage outside the ranges of the input variables.

An ANN is a highly interconnected system inspired by the brain and formed by simulated “neurons” represented by a transfer function, and “weights” associated to the connections of the “neurons.” The back propagation training algorithm allows experimental acquisition of input/output mapping knowledge within multilayer networks. Because we have experimental data on the cumulative numbers of pits versus time of observation, as a function of oxidation state, minor alloying element, and applied potential, we decided to use an ANN backward propagation technique with supervised learning. During training of the ANN, the cumulative numbers of pits were used as “output” and the applied potential, oxidation state, minor alloying element concentration, and time of observation as “inputs.” We explored several topologies to obtain the best compromise between learning and computing time for an ANN with 2 hidden layers.

The maximum training time was set to 12 hours on a Macintosh II microcomputer with a threshold of 10% of the normalized input values (input-output) [2].

The ANN had the following features:

- Heteroassociative memory, for which the patterns on recall from the memory are purposely different from the input pattern, because the inputs and outputs are different and belong to different classes of information.
- Delta rule type of learning, where the neuron weights are modified to reduce the difference between the desired output and the actual output of the processed element. The weights are changed in proportion to the error calculated. This rule also limits the learning, if the error at the output of the network is lower than a given threshold. The learning rates of those layers close to the output are set lower than the learning rates of the other layers.
- A momentum term, which is used to smooth out the changes.
- A sigmoid transfer function, which is a monotonically continuous mapping function.

**Fig. 5:**
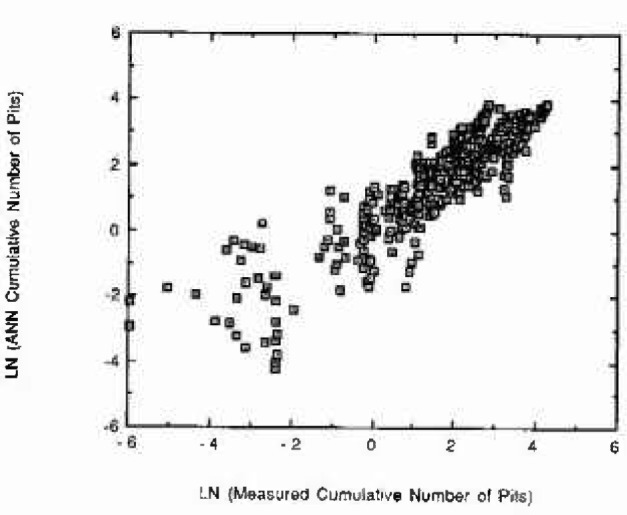
Natural logarithm of the predicted ANN total number of pits versus natural logarithm of laboratory measured total number of pits. The measurements included several: applied potentials, observational times, oxidation states, and percent of minor alloying elements.

The ANN predictions are in good agreement with the measured data. Figure 5 shows that correlation. Considering the difficulty of obtaining high quality data, we consider that the correlation factor is satisfactory.

**Fig. 6:**
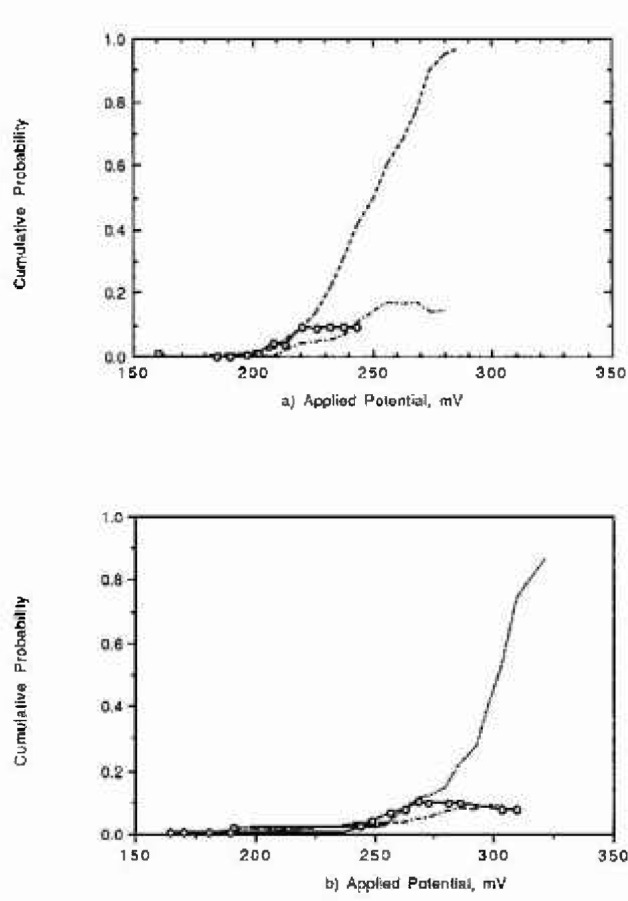
ANN prediction of cumulative number of pits versus applied potential, at 50 s time of observation; and several percentiles of minor alloying elements. (6a) Oxidation state (4-2). (6b) Oxidation state (6-2).

After the ANN was trained, it was used to make predictions of the number of pits at different applied potentials, observation times, oxidation states, and percentages of minor alloying elements in the film. The total number of pits predicted by the ANN decreased with increasing percentage of minor alloying elements in the film, and with increasing oxidation state of the alloying element (Figs. 6a and 6b). Behavior similar to that predicted by ANN was observed experimentally.

The ANN, once trained, can be used to explore the importance of the relationship between “output” and “inputs.” We found that the results were strongly dependent on observation time (*t*^3^, *t*), have a medium dependency on applied potential (*V*^1/2^), and show weak dependencies on oxidation state (*Z*^1/3^) and concentration of minor alloying element in the film ([%]^1/4^).

## 6. Discussion

**Fig. 7:**
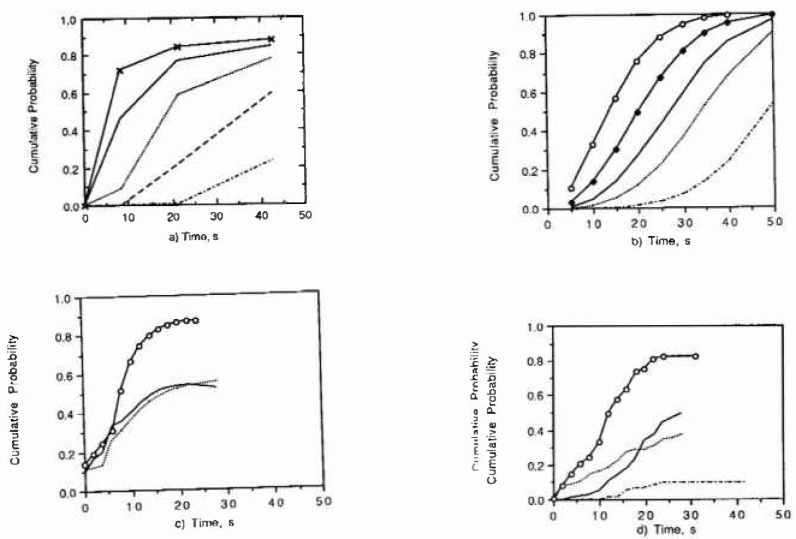
Cumulative number of pits versus time of observation at several applied potentials; Oxidation state (3-2); and 3% of minor alloying clement. (7a) Predictions using the deterministic model. (7b) Predictions using Weibull Distribution model with 2 fitting parameters. (7c) Predictions using the ANN. (7d) Laboratory measurements.

Figures 7a to 7d show the hest predictions obtained with the three models compared with the laboratory data.

The deterministic model predicts that the cumulative probability at low applied potentials is not only described by a flat curve but that the curve is displaced to higher times. This prediction coincides with the experimental observations. The predictions with the deterministic model at high potentials indicated that the plateau corresponding to higher times is reached sooner than that measured. The deterministic model is the only model (compared to the other two models) that brings together an understanding of the problem as well a predictive tool. Another advantage that the deterministic model has over any nondeterministic model is that to fit the model, only an experimental datum point is necessary to calibrate the model to the data. The deterministic model was developed to predict damage and cumulative number of pits simultaneously. This last capability makes it very attractive to the user.

The results obtained with the probabilistic model are in general agreement with the experimental observations. As with the deterministic model, the plateau in cumulative damage is reached sooner than the measured one. However, the curves are flatter at lower potentials than at high potentials, but they are not displaced to higher times. The probabilistic approach needs a large data base, and the predictive capabilities are limited to the ranges of variables confined in the data base.

The ANN model describes the cumulative number of pits very close to the experimental measurements. The plateaus on cumulative damage correspond very well to the plateau obtained experimentally. The ANN predictions at low number of pits are inaccurate, but they are very close to the experimental observation at higher cumulative numbers of pits.

We conclude that little is learned about the phenomena when nondeterministic models are used; however, they can represent invaluable predictive tools. The statistical model is in a sense more de manding that an ANN model. It requires a robust and large data base. We found that an AIIN can learn from “noisy” data, and that the range at prediction can be extended outside the range for which it was trained, if trained correctly [13]. Damage functions can also be calculated using I he deterministic model based on the PDM and SVIM. The deterministic model does not need to have an extensive data base that includes pit depth distributions. On the other hand, the nondeterministic models need a large data base, and they are unable to make predictions of partial damage if the pit depth versus number of pits is not confined to the data base. Cumulative damage can be interpolated and extrapolated to other voltages and times, and to other applied potentials, for any of the three models, In general the results obtained with the three models were found to be in reasonable agreement with experimental data [12].

We do not intend to emphasize here the importance of deterministic models over nondeterministic models, but we have to keep in mind when picking a model, to choose the. one that best represents the observations and that is reasonably easy in solve. Clearly, the reliability of the extrapolation. in particular, depends critically on the quality of the data and on the veracity of the model

## Figures and Tables

**Fig. 3a:**
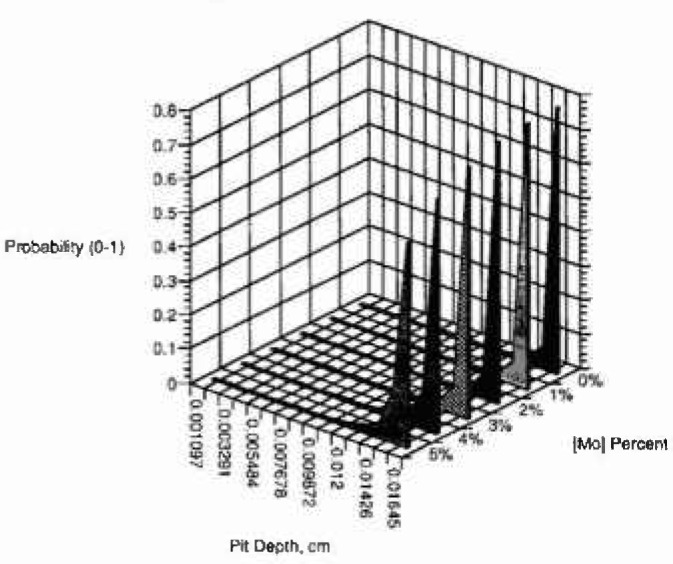
Cumulative probability, calculated using the deterministic model, versus pit depth for several concentrations of minor alloying clement segregated in the film for oxidation state (3-2).

**Fig. 3b:**
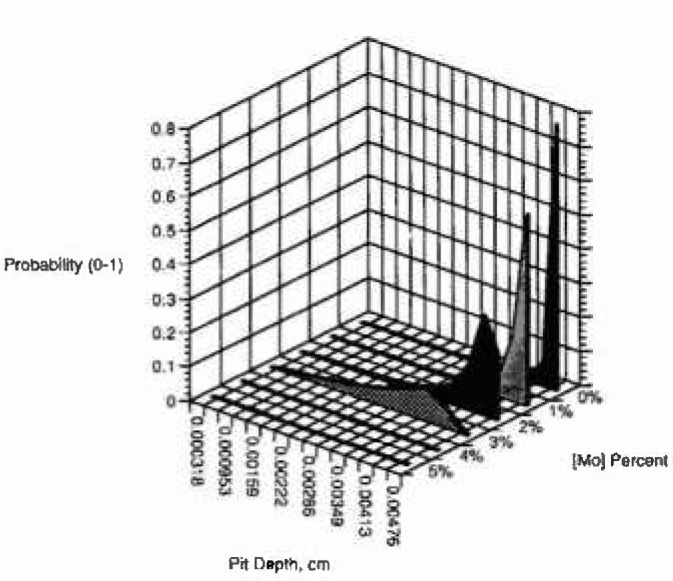
Cumulative probability, calculated using the deterministic model, versus pit depth for several concentrations of minor alloying clement segregated in the film for oxidation state (4-2).

**Fig. 3c:**
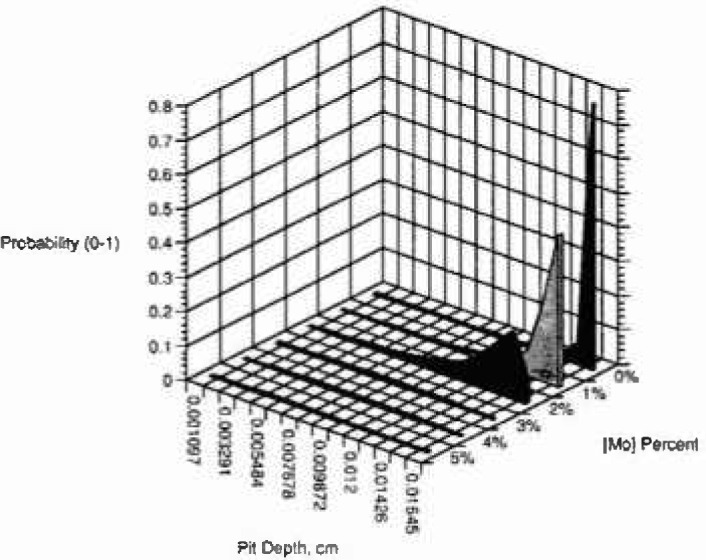
Cumulative probability, calculated using the deterministic model, versus pit depth for several concentrations of minor alloying element segregated in the film for oxidation state (6-2).

**Table 1:** Input data used in the calculation of the deterministic/probabilistic model

Parameters	Value	Units
Stoicbiomctry	2	
Avogrado constant	6.023 E + 23	mol^−1^
Mol vol. of oxide cation	30	cm^3^/mol
Gibbs energy change^a^	−40,000	J/mol
Gibbs energy change^a^	−10,000	J/mol
Mean diffusion coefficient	5 E−20	cm^2^/s
Standard deviation	0.75 Dmcan	cm^2^/mol
Chloride activity	0.573/2	
Electrical field across film	1.1 E + 6	V/cm
Alpha	0.65	
Beta	−0.01	V/pH unit
Critical area vacancy size^a^	1 E+16	No/cm^2^
Critical vacancy flux^a^	15.87 E+12	No/cm^2^
Temperature	298.15	K
Applied potential	−0.55	V SHE
Molar gas constant	8.314	J K^−1^mol^−1^
Electrical potential film/sol	−0.5	V SHE

a Variables that were used to adjust one datum point to scale the results properly.
